# Can third-party observers detect attraction in others based on subtle nonverbal cues?

**DOI:** 10.1007/s12144-022-02927-0

**Published:** 2022-04-08

**Authors:** Iliana Samara, Tom S. Roth, Milica Nikolic, Eliska Prochazkova, Mariska E. Kret

**Affiliations:** 1 Cognitive Psychology Unit, Department of Psychology, Leiden University, Wassenaarseweg 52, 2333 AK Leiden, the Netherlands; 2 Leiden Institute for Brain and Cognition (LIBC), Leiden, the Netherlands; 3 Apenheul Primate Park, Apeldoorn, the Netherlands; 4 Research Institute of Child Development and Education, University of Amsterdam, Amsterdam, the Netherlands

**Keywords:** Attraction, Emotion detection, Third-party observer, Zero-acquaintance, Speed-dating

## Abstract

In a series of three studies, we examined whether third-party observers can detect attraction in others based on subtle nonverbal cues. We employed video segments of dates collected from a speed-dating experiment, in which daters went on a brief (approx. 4 min) blind-date and indicated whether they would like to go on another date with their brief interaction partner or not. We asked participants to view these stimuli and indicate whether or not each couple member is attracted to their partner. Our results show that participants could not reliably detect attraction, and this ability was not influenced by the age of the observer, video segment location (beginning or middle of the date), video duration, or general emotion recognition capacity. Contrary to previous research findings, our findings suggest that third-party observers cannot reliably detect attraction in others. However, there was one exception: Recognition rose above chance level when the daters were both interested in their partners compared to when they were not interested.

**Supplementary Information:**

The online version contains supplementary material available at 10.1007/s12144-022-02927-0.

Humans swiftly produce and infer emotional states through facial or bodily expressions in everyday life. Even though some emotional states might be easier to recognize than others (e.g., happiness, Camras & Allison, 1985), humans can efficiently communicate their emotional state using nonverbal cues even in as little as 3 s (Meltzer et al., 2019). A crucial emotional state regarding mate choice, yet commonly misinterpreted, is *attraction* (e.g., Farris et al., 2008; Haselton & Buss, 2000). Observing and decoding subtle nonverbal cues, such as blushing or a faint smile, might facilitate answering whether a person would be interested in seeing another again; however, whether such nonverbal cues can be accurately detected as efficiently as other emotions has not yet been examined. In the present study, we investigated whether third-party observers could detect attraction between strangers during a speed-date using thin video slices.

Attraction is a powerful emotion. It can guide our behaviour during social interactions, pulling us towards people we find attractive or interesting (Montoya & Horton, 2020). Like other emotions (e.g., anger or fear), attraction influences others' behavior (e.g., Ekman, 1992; Montoya et al., 2018; Russell, 2003). Notably, the experience of attraction is linked to heightened arousal, which previous research has demonstrated by measuring these psychophysiological processes via heart rate and electrodermal conductance (Foster et al., 1998; but see Prochazkova et al., 2021). These physiological processes can act as somatic markers (Damasio, 1996) and are used in efficiently interpreting an ambiguous situation, such as a first romantic encounter. Interestingly, previous research has shown that people on a speed date can indicate whether they would like to meet their partner again only after 3 s of looking at their partner, and their judgment remains (mostly) consistent throughout the speed date (Prochazkova et al., 2021). Thus, these findings illustrate that attraction can emerge quickly, linked to specific physiological processes, and guide behaviour during social interactions.

Humans might often hide their feelings or convey the opposite to steer social interactions in the desired direction (Kret, 2015). However, despite our best efforts to control our emotional expressions, there are specific cues over which we have no control (Grammer et al., 2000; for a review, see Prochazkova & Kret, 2017). For example, upon viewing someone that interests us, our pupils might dilate, and a distinctive blush might appear on our face (Eibl-Eiblsfeldt, 1989; Keltner & Buswell, 1997). Indeed, nonverbal cues primarily communicate attraction (Givens, 1978). The vast repertoire of expressions encapsulating attraction and how they are expressed have prompted the term “courtship dance” (Birdwhistell, 1970). Multiple signals reflecting attraction have been catalogued, even if the senders might not always be aware of producing them (Grammer et al., 1998; McCormick & Jones, 1989; Moore, 2010). Coy smiles, genuine smiling, blushing, hair flipping, leaning forward, rolling the pelvis, and head tilting are a few of the signals listed in previous research (Argyle, 1988; Eibl-Eiblsfeldt, 1989; Givens, 1978; Grammer et al., 2000; Moore, 1985, 2010). Therefore, even if there is no clear-cut expression, there are subtle nonverbal signals that, when expressed, indicate interest and availability.

Emotional expressions are not only sent to others, however, but they also need to be efficiently interpreted for them to be informative and useful. However, a given signal can often be ambiguous. This ambiguity is similar to a verbal exchange, where one statement can be interpreted in multiple ways by the perceiver, who is tasked with inferring the statement's message (for a comprehensive review, see Vangelisti, 2015). It is important to note that it might be easier to detect attraction in a later phase of a speed date than during a first impression (e.g., Place et al., 2009). This is not surprising, given that beginning of dates is typically more stilted than later during the interaction. During a first impression, it is typical that people are more reserved and do not display as many nonverbal behaviours as they typically would, perhaps to reduce the likelihood of rejection or to adhere to social norms (Kunkel et al., 2003). This might translate into people being better able to detect the absence of attraction rather than its presence, as shown in a recent study (Hall et al., 2015). In that study, participants watched six one-minute videos of people on a date (only one person from the couple; 3 men and 3 women). Participants indicated, amongst other items, whether the person depicted was flirting with their partner (yes/no). Accuracy was coded as a match between the participants' and the daters' responses. The results showed that participants were more accurate in detecting (the absence of) attraction when the daters were not flirting than the presence of attraction when the daters were flirting. The authors argued that since base rates of flirting behaviours in zero-order acquaintance settings are low, people might lack knowledge of cues reflecting attraction to detect and interpret them efficiently. These findings suggest that it is challenging to detect attraction in others during first impressions since behaviours signaling attraction are not typically displayed.

Notably, previous research typically utilized videos of dates as stimuli and asked third-party observers to indicate whether the people involved in the date were attracted to each other or not (e.g., Hall et al., 2015; Place et al., 2009). However, factors such as the angle and distance of the camera from the people might have made it challenging to observe minute emotional expressions (for instance, a faint or coy smile), which would have facilitated gauging the others' interest. In contrast, in other previous work (Prochazkova et al., 2021), participants were filmed in close range, so subtle spontaneous emotional reactions are easy to detect. Therefore, an uninvolved third-party observer might be able to decode attraction cues better than the persons in the date themselves if the date allowed for less stilted behaviours and if subtle expressions were expressed and visible. Furthermore, despite previous research showing both daters simultaneously (e.g., Hall et al., 2015; Place et al., 2009, 2012), the effect of synchronous behaviour between the daters has not been directly examined. Indeed, mimicry has been shown to increase the chance of liking and affiliation with others (Chartrand, & Bargh, 1999; Cheng & Chartrand, 2003; Lakin & Chartrand, 2003; see also Roth et al., 2021a, 2021b). Therefore, if synchronous behaviour between two daters facilitates the detection of attraction (i.e., dater A smiles and dater B reciprocates that smile), then the presentation of randomly shuffled videos would impair accuracy in detecting attraction. Thus, two factors that might influence accuracy in detecting attraction in others, namely subtle expressions and synchronous behaviour, have not been disentangled in previous research.

Many factors might influence detecting attraction in others (Place et al., 2009). Place et al. (2009) examined the possible effects of age on attraction accuracy. In their adult sample, they found no evidence that age mattered. However, the age range of their sample was limited to young adults (18–27 years old). Thus, whether age influences accuracy in detecting attraction when including a wider range remains unclear. Nevertheless, there is a reason to assume that age may influence detecting attraction. First, young individuals, specifically children, will have less relationship experience than adults. Given that such experience is essential for detecting and interpreting emotions according to the Perception–Action Model of Empathy (PAM; de Waal & Preston, 2017), adults, who are more experienced with romantic attraction, should detect attraction in others considerably better than children. Second, brain areas important for emotion expression processing are still under development in children (Thomas et al., 2007). Thus, younger children have more difficulties recognizing emotions than older children and adults, especially when the emotions are complex (Pons & Harris, 2005) or social and subtle (Thomas et al., 2007). Third, attraction may not be evolutionary relevant for young children before they enter puberty and become interested in sexuality (Baams, Dubas, Overbeek, & van Aken, 2015). It is, thus, more likely that children become better at detecting attraction with age.

Here, in a series of three experiments, we examined whether third-party observers could detect attraction between strangers on a date after observing only thin slices of that interaction (i.e., 3–9 s). Specifically, we examined whether this is influenced by a) age (Experiment 1 and Experiment 2) or the interaction phase (i.e., first impression or middle of the date) and stimulus presentation duration (Experiment 3); and b) when the person observed is indeed interested in their partner than when not. To investigate these hypotheses, we asked participants to indicate whether the daters would like to go on another date with their partner, which was considered a proxy for attraction. Previous evidence has shown a moderate correlation between physical attraction and the likelihood of wanting to meet a partner again (Veenstra & Hung, 2011). We expected that third-party observers would be significantly more accurate than chance level in detecting attraction, given the plethora of subtle expressions visible in the video segments. Based on previous findings (Hall et al., 2015), we also aimed to examine whether detecting attraction is facilitated as a function of whether the person depicted is interested in their partner or not. Hall et al. (2015) found that lack of attraction is easier to detect. However, if people were interested in their partner, they might produce more salient and interpretable cues than not, resulting in increased attraction detection accuracy.

## General methods

### Stimuli

**Fig. 1:**
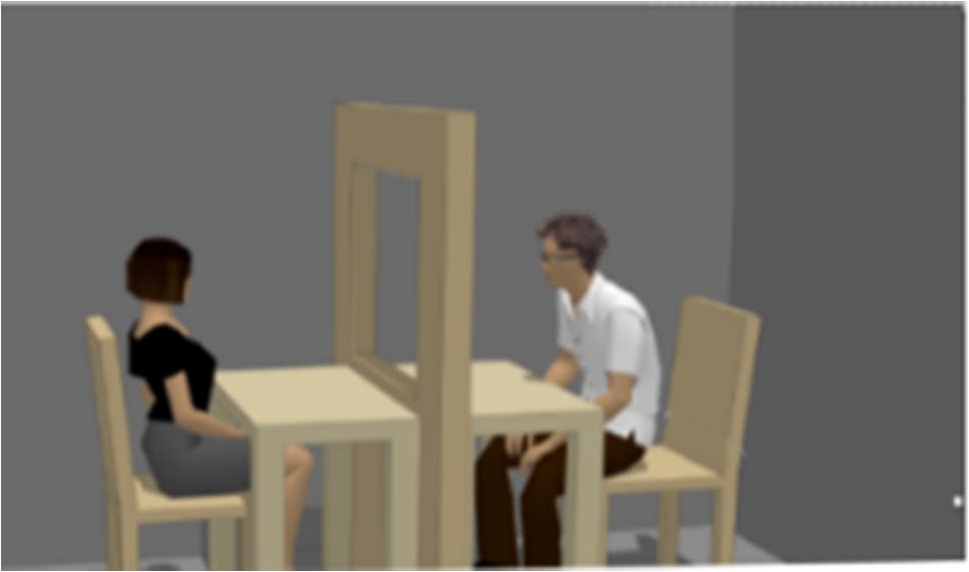
Experimental setup of Prochazkova et al. (2021). Reprinted with permission

Stimuli consisted of muted video fragments collected during a blind date study conducted at the Lowlands festival (Lowlands, the Netherlands) (Prochazkova et al., 2021). In that study, participants were seated at opposite ends of a table with a barrier blocking their partner from view (see Fig. 1). Participants were informed that they would have three separate interactions with their partner: a first impression phase (FI; 3 s), an eye contact phase (EC; 2 min), and a verbal interaction phase (VI; 2 min). The EC and VI phases were counterbalanced across couples. During the FI phase, the barrier was lifted, and participants saw each other in a flash of 3 s and were not allowed to speak. The barrier was then lowered, obscuring the partners from view. During the VI phase, the barrier was lifted, and participants were allowed to communicate with each other for 2 min. During the EC phase, the barrier was lifted, and participants were not allowed to communicate with each. The barrier was lowered again between the VI and EC phases. Video was recorded using the Tobii wearable eye-tracker glasses (Tobii Sweden), meaning that the video of each participant reflects the first-person perspective of their partner. In our study, all stimuli started as soon as the barrier was fully lifted and continued for 3, 6, or 9 s. Only stimuli from the FI (Study 1, Study 2, Study 3) and VI (Study 3) were used in the present study. All stimuli were shown against a grey background. Out of the 32 videos used, 16 depicted individuals (10 men and 6 women) that indicated that they were interested in their partner (50% base rate across all individuals). As a manipulation check, we examined differences in frequency and duration of behaviors signaling attraction between daters attracted to their partner compared to daters that were not attracted to their partner for FI and 9-s VI stimuli. The results showed that in 9-s VI stimuli, participants interested in their partner showed a greater duration of such behaviors, such as coyness, than participants who were not interested in their partner (see Supplemental Material).

### (Current) experimental task

**Fig. 2:**
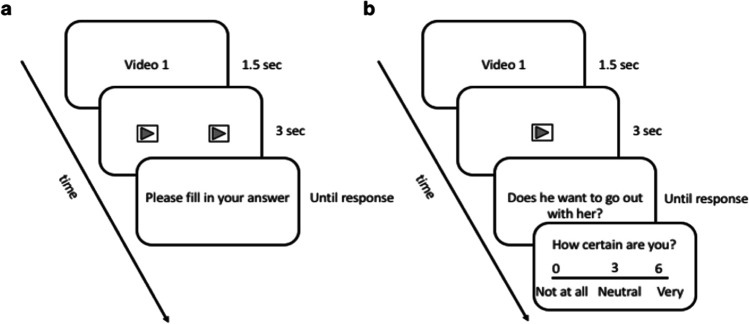
Progression of a typical trial in the experimental task for Experiment 1 (a) in which both members of a couple were presented simultaneously, and responses were logged using pen and paper and Experiment 2 (b), where only one member of the couple was displayed. The question in figure b is an example for only the male couple member presented, the questions were formed depending on the couple member’s gender

The experimental task was controlled by a script written in E-Prime (Version 2; Psychology Software Tools, Pittsburgh, PA). Figure 2 illustrates the progression of a typical trial in the task. Each trial started with a screen showing the trial number (1.5 s), followed by the presentation of the videos either in a side-by-side (woman left) or one-by-one fashion (3 s). Participants were instructed to attend to the videos presented, with no specific instructions regarding which video they should attend to specifically. Another screen followed on which participants were asked to indicate whether the person(s) they viewed would like to go on another date with their partner (separately for the male and female couple member displayed: “Does he want to go on another date with her?” “Does she want to go on another date with him?”) and remained on the screen until an answer was provided (in Experiment 1, responses were provided using a pen and paper questionnaire). Finally, a screen appeared on which participants were required to indicate their degree of certainty regarding the previous response, which also remained on the screen until an answer was provided.

### Statistical analyses

We computed accuracy scores by comparing the participants’ responses (0 = no; 1 = yes) with the actual responses of the members of the dating couples. These accuracy scores (0 = incorrect; 1 = correct) were analyzed using Bayesian logistic multilevel modeling (MLM). Bayesian MLM's use allowed us to both account for the dependence in our data and quantify the support for either the null or alternative hypothesis present in our data (Jeffreys, 1961; Lee & Wagenmakers, 2014; van Doorn et al., 2019). All our analyses consisted of three models, each testing a separate hypothesis. We conducted an intercept-only model to examine whether people can accurately detect attraction. In the second model, we examined whether accuracy differs as a function of age or the video segment properties by including the fixed effect of Group or Video Condition for Experiments 1–2 and Experiment 3, respectively. In the third model, we examined whether the accuracy of the uninvolved third-party observers was enhanced when the daters themselves were attracted to their partner by including the fixed effect of Attraction to Partner. Additionally, in Experiment 1, we examined whether synchronous behaviour between the daters influences accuracy by including the fixed effect of the Shuffled condition. All our models included a random effect of Participant. The minor adjustments due to the factors present in each experiment design are further explained in the corresponding statistical analyses section.

There has been a long-standing debate about optimal priors for logistic models (e.g., Christensen et al., 2011; Gelman et al., 2007). Uniform priors can exert undue influence on the posterior distribution of the underlying parameter (McInturff et al., 2004) and therefore, weakly informative priors are better suited (Seaman et al., 2012). We have opted to use a normal distribution for all input values despite previous literature pointing towards Cauchy priors (Gelman et al., 2007; Ghosh et al., 2018). All priors were centered at 0 and had a standard deviation of 1 for all coefficients except the constant (*SD* = 0.8). We further included an exponential prior (*SD* = 1) to all error terms. Finally, binary inputs were sum-coded (-1 vs. 1).

The interpretation of Bayesian logistic MLM estimates might not be intuitive. Therefore, we report multiple estimates to illustrate the robustness and uncertainty of an effect (e.g., see Martin et al., 2020). The median estimate coefficient is reported together with the 95% Highest-Density Credible Intervals (*HDI*), which summarize the posterior parameter values with the highest probability density (Kruschke, 2018). Furthermore, we report the probability of direction (*pd*), the proportion of the probability in support of a hypothesized positive or negative effect (Makowski et al., 2019). To examine the robustness of interactions, we performed model comparisons to calculate Bayes Factors (*BF*). BFs are interpreted following the scheme of Jeffreys (1961), who suggested BF values 0–3 to be considered anecdotal evidence, 3–10 moderate, and greater than ten strong evidence in favour of either the alternative (*BF*_10_) or null (*BF*_01_) hypothesis.

Model convergence was examined using the guidelines detailed in the WAMBS checklist (Depaoli & van de Schoot, 2017). Specifically, for every model, we examined the Gelman-Rubin diagnostic values (a value close to 1 indicates convergence), as well as trace, autocorrelation plots, and density histograms for all posterior distributions. Analyses were conducted in R (version 3.6.1; R Core Team, 2019) using the brms package (Bürkner, 2017, 2018).

## Experiment 1

### Methods

#### Participants

Sixty-one adults (*n* = 61; age range: 18—54; *M* age: 26.13, *SD* = 6.40; 42 female) and 60 children (2 excluded for inattentiveness, final *n* = 58, age range: 8—14; *M* age: 10.00, *SD* = 1.63; 25 female) were recruited during a science festival (Rotterdam, the Netherlands). The sample size was determined by the number of people that wanted to participate during this event and is comparable to the studies by Place et al. (2009). All participants provided informed consent and were informed that they could withdraw their participation with no adverse consequences as according to the Declaration of Helsinki. For children younger than 12 years old, consent was provided by their parents, whereas for children older than 12 years old, consent was provided by both the parents and the children. The study was approved by the Leiden University Psychology Ethics Committee (CEP19-0424/290). Participants were not remunerated for their participation.

#### Stimuli

**Fig. 3:**
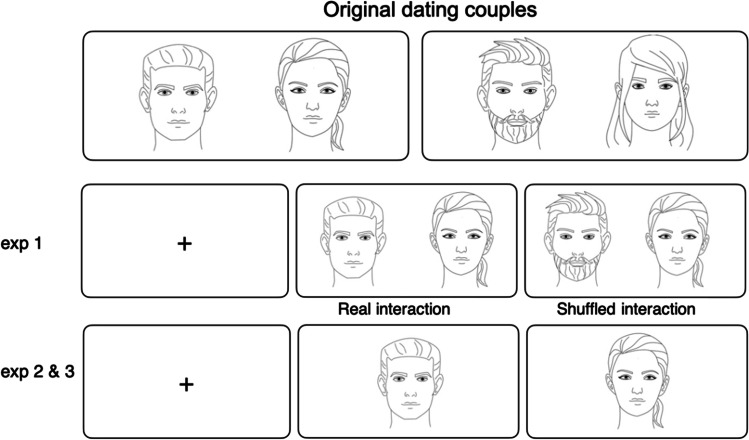
Stimuli and stimulus progression *Procedure*

Stimuli consisted of videos of the couple members during the first impression (FI) presented side by side on the display for 3 s (see Fig. 3). The 3-s videos were selected as in the original study, participants could report within 3 s whether they were interested in their partner, and crucially, their responses remained relatively consistent throughout the speed date (Prochazkova et al., 2021). The original videos (i.e., with background) were displayed. To examine the effect of synchrony on the detection of romantic interest, we manipulated the presentation of interactions in the couples (i.e., Shuffled condition). Specifically, half of the couples (*n* = 8) were not shuffled and were presented as collected (henceforth known as *real* interaction). In contrast, the rest of the videos were randomly shuffled and presented to create fake interactions that actually never took place (e.g., see Fig. 3: bearded man dated the woman wearing her hair down but was presented in the Shuffled condition next to the woman wearing her hair in a ponytail). This factor was implemented as a control to ensure that it is specific cues of the person not necessarily the interaction between the couple that influenced the participants’ response.

Participants were asked to provide informed consent. Next, they were seated in front of a computer monitor (25-inch, 1680 × 1050 resolution; 60 Hz refresh rate). Participants filled in their answers for each trial in a paper questionnaire. The task consisted of 16 trials and lasted approximately 5 min. After the end of the task, participants were debriefed and thanked for their participation.

#### Statistical Analyses

We performed all analyses as detailed in the Statistical Analyses [General Methods section] with the following adjustments: To account for the fact that the videos were shuffled, we included the fixed effect of Shuffled and its interaction with Age Group and Attraction to Partner in Models 2 and 3, respectively. Furthermore, given that participants performed the study in the presence of other participants, we recorded their subgroups (*GroupID*) and included a random intercept per Participant nested in *GroupID*.

### Results

**Fig. 4:**
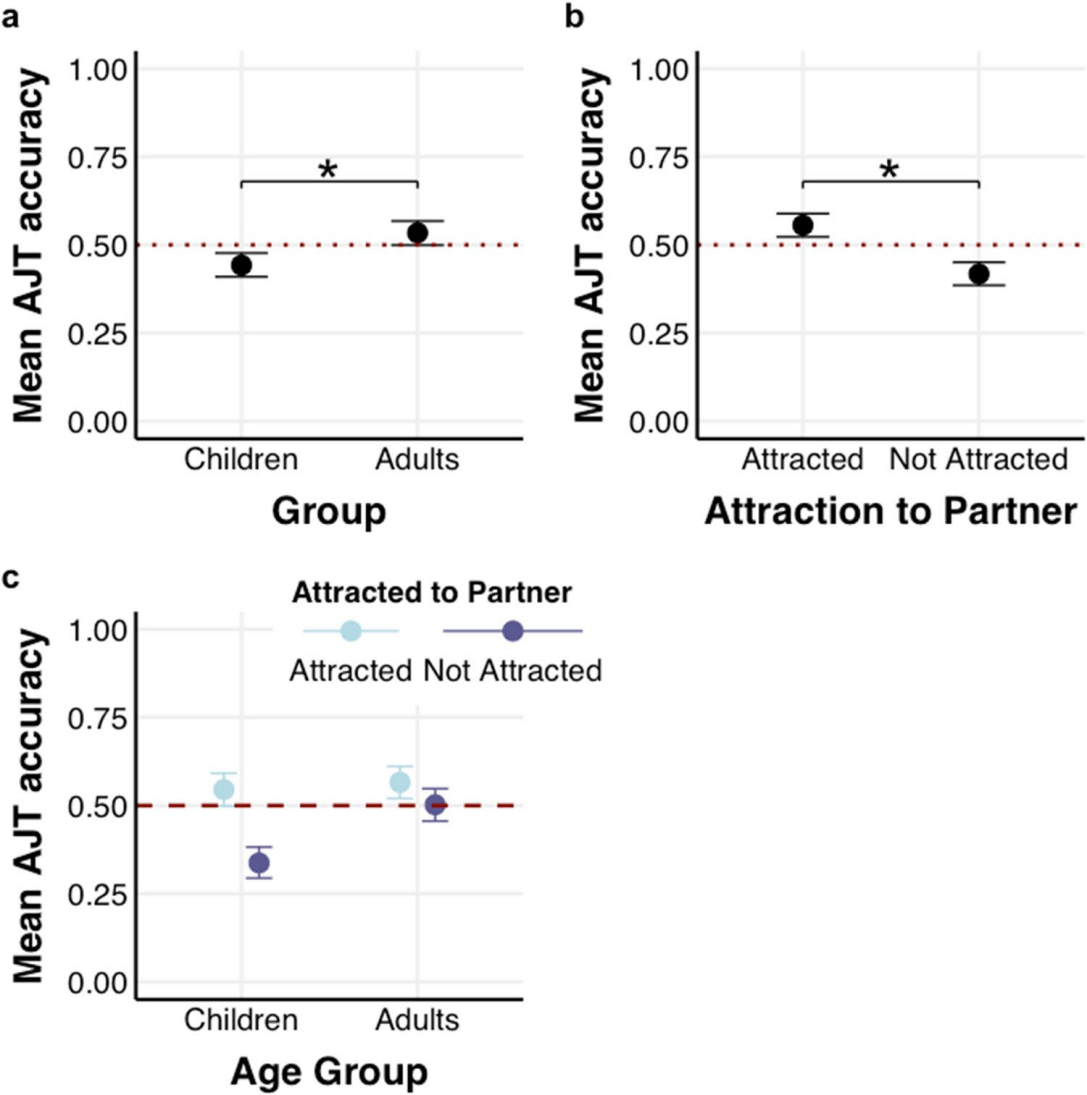
**(a)** Mean accuracy in the Attraction Judgment Task (AJT) as a function of Group (Children vs Adults). The graph shows that children performed below chance level (0.5), whereas adults did not differ from chance level; **(b)** Accuracy as a function of Attraction to Partner (Attracted vs Not attracted). The graph shows that participants performed above chance level (0.5) when the person depicted was attracted to their partner compared to when they were not. **(c)** Accuracy as a function of Attraction to Partner (Attracted vs Not attracted) and Age Group (Children vs Adults). The graph shows that children performed worse when the person depicted was not attracted to their partner. The red line denotes chance level (0.5) and all error bars reflect 95% Credible Intervals (CrI)

All models are presented in Table 1. Contrary to our hypothesis, we did not find robust evidence that participants could detect attraction overall (*β* = -0.06; [-0.15, 0.02]; *p*_-_ = 93.62%). Our hypothesis that age would influence the detection of attraction was confirmed: the model showed that children performed worse than adults (*β* = -0.14, 95% HDI [-0.21, -0.07], *p*_-_ = 99.99%); however, adults did not substantially differ from chance level (i.e., 0.5; see Fig. 4a). There was no substantial difference in accuracy as a function of Shuffled (*β* = -0.01, 95% HDI [-0.07, 0.06], *p*_+_  = 60.64%) or an interaction between Shuffled and Age Group (*β* = 0.04, 95% HDI [-0.02, 0.10], *p*_+_  = 90.76%; BF_01_ = 12.59) indicating that synchrony did not influence accuracy in detecting attraction.

**Table 1:** Overview of all accuracy predicting models (1–3) for Experiment 1

Predictors	Accuracy (Median estimate of the coefficient with 95% HDI)		
	**Model 1**	**Model 2**	**Model 3**
	β (95% HDI)	β (95% HDI)	β (95% HDI)
Intercept	-0.06 [-0.15, 0.02]	-0.06 [-0.13, 0.01]	-0.06 [-0.13, 0.01]
Age Group		-0.14 [-0.21, -0.07]	-0.15 [-0.22, -0.07]
Shuffled		-0.01 [-0.07, 0.06]	-0.01 [-0.07, 0.05]
Attracted to Partner			0.36 [0.29, 0.42]
Age Group × Shuffled		0.04 [-0.02, 0.10]	0.04 [-0.02, 0.11]
Age Group × Attracted to Partner			0.14 [0.07, 0.20]
Shuffled × Attracted to Partner			0.08 [0.02, 0.15]
Age Group ×			-0.01 [-0.08, 0.05]
**Random Effects**			
Var(Group ID)	0.02	0.00	0.01
Var(Participant)	0.00	0.00	0.00

To examine whether participants can detect the presence of attraction, we included the fixed effect of Attraction to Partner and its interaction with Age Group and Shuffled. The model showed that participants were more accurate when the person in the video indeed was attracted to their partner than when they were not (see Fig. 4b; *β* = 0.36; 95% HDI [0.29, 0.42], *p*_+_  = 100%). Children performed worse than adults (*β* = -0.15, 95% HDI [-0.22, -0.07], *p*_-_ = 100%). There was no substantial difference between real and shuffled videos (Table 1; Model 2; *β* = -0.01; 95% HDI [-0.07, 0.05]; *p*_+_  = 59.55%), or an interaction between Shuffled × Age Group (*β* = 0.04, 95% HDI [-0.02, 0.11], *p*_+_  = 89.80%; BF_01_ > 10). The interaction between Group and Attraction to Partner was reliable (see Fig. 4c*; β* = 0.14, 95% HDI [0.07, 0.20], *p*_+_  = 100%; BF_10_ > 10); indicating that children performed worse when the daters depicted were not attracted to their partner compared to when they were attracted to their partner. The interaction between Shuffled and Attraction to Partner was not reliable (*β* = 0.08, 95% HDI [0.02, 0.15], *p*_+_  = 99.22%; BF_10_ = 0.68), as well as the interaction between Shuffled, Attraction to Partner, and Age Group (*β* = -0.01, 95% HDI [-0.08, 0.05], *p*_-_ = 66.25%; BF_01_ > 10). For that reason, these interactions are not interpreted.

### Discussion experiment 1

In Experiment 1, we aimed to examine if a) people accurately detect attraction; b) whether this ability is influenced by Age Group (as an index of experience); c) synchrony between daters; and d) whether accuracy is enhanced when the daters themselves were interested in their partner. The results of Experiment 1 showed that participants overall did not detect attraction or the absence of it better than chancel level (0.5). Regarding our second hypothesis, we found that children performed below chance level. Crucially, we found that videos in which couple members were attracted to their partner were detected more accurately than ones in which they were not. Synchrony between daters did not seem to influence the ability to accurately detect attraction in others.

A possible explanation for the low accuracy observed could be that attending to the videos required dividing attention over two separate video streams (one for the male and one for the female). This division of attention combined with the brief duration of the video segments (3 s) might have impaired efficient processing of our stimuli. Indeed, previous research has shown that dividing attention has a negative effect on decision making (e.g., McCrink & Hubbard, 2017 for operational momentum). Therefore, in Experiment 2, we simplified our experimental procedure by presenting stimuli one-by-one.

## Experiment 2

The results of Experiment 1 showed that synchrony does not influence the accuracy of participants in detecting whether daters were attracted to their partner or not. Therefore, in this experiment, we presented the same stimuli as in Study 1, with the sole difference that only one couple member was presented in every trial so as to reduce cognitive load. This adjustment allowed us to examine whether reduced cognitive load would enhance accuracy in detecting attraction. Furthermore, participants performed the experimental task on a personal laptop.

### Methods

#### Participants

Thirty-eight adults (age range: 18—66; *M* age: 40.40, *SD* = 15.30; 21 female) and 26 children (age range: 8—12; *M* age: 9.80, *SD* = 1.40; 12 female) were recruited during the Afternoon and Night of Discoveries event (Leiden, the Netherlands), respectively. All participants provided informed consent and were informed that they could withdraw their participation with no adverse consequences as according to the Declaration of Helsinki. The study was approved by the Leiden University Psychology Ethics Committee (CEP19-0722/418). Participants were not remunerated for their participation. Differences in participants’ age and gender between Experiment 1 and Experiment 2 are reported in the Supplementary Material.

#### Procedure

After participants provided informed consent, they were invited into the experimental cabin and seated in front of a Dell laptop (15-inch display; 60 Hz refresh rate). Instructions were presented on display and also explained by a researcher. Participants were informed that they would view a series of videos and indicate whether the person depicted would like to go on another date with their partner or not and their level of certainty regarding their judgment. Participants were instructed to respond as fast and accurately as possible. To limit distraction, participants wore noise-reduction earmuffs throughout the task.

Participants were prompted to indicate whether the person would like to go on another date with their partner by pressing the corresponding keyboard key (*j/y* for ja or yes, and *n* for no); followed by their certainty regarding their decision from 0 (*not at all*) to 6 (*very*) with 3 indicating neutral level of certainty. The task consisted of 32 trials in total and lasted approximately 5 min.

#### Statistical Analyses

Trials with RTs < 200 ms were excluded (0.25% adults’ dataset; 0.24% children's data; Whelan, 2008). We followed the same modeling steps as in Experiment 1 with the only difference that the random intercept per participant was not nested in GroupID (since there was no such factor in the current design).

### Results

**Fig. 5:**
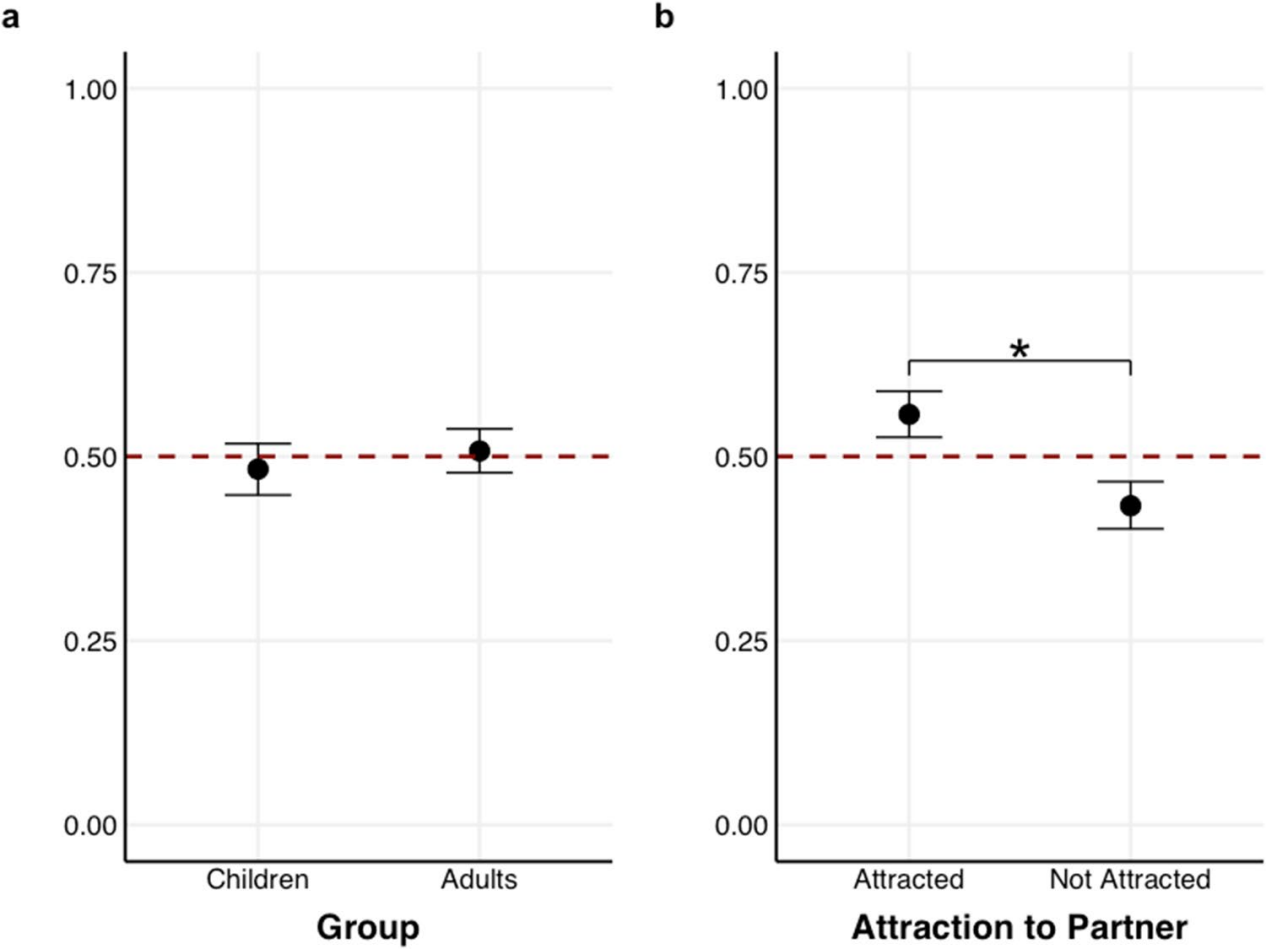
**(a)** Accuracy as a function of Group (Children vs Adults). The graph depicts that both children and adults performed at chancel level (0.5); **(b)** Accuracy as a function of Attraction to Partner (Attracted vs Not attracted). The graph depicts that participants performed above chance level (0.5) when the person depicted was attracted to their partner compared to when they were not. The red line denotes the chance level, and all error bars reflect 95% Credible Intervals (CrI).

First, we did not find substantial evidence that participants could reliably detect attraction (*β* = -0.01; 95% HDI [-0.10, 0.08], *p*_-_ = 56.62%). To examine our second hypothesis, we included the fixed effect of Age Group. The model showed that accuracy did not substantially differ as a function of Group (*β* = -0.05; 95% HDI [-0.14, 0.04]; *p*_-_ = 85.70%). Next, we modeled participants’ accuracy by including the fixed effect of Attraction to Partner and its interaction with Age Group. As in Experiment 1, the model showed that participants were more accurate when the person in the video indeed was attracted to their partner than not (*β* = 0.25; [0.16, 0.34]; *p*_+_  = 100%; see Fig. 5*;* Table 2, Model 3). Accuracy did not differ as a function of Age Group (*β* = -0.05; 95% HDI [-0.14, 0.04]; *p*_-_ = 86.25%). The interaction between Age Group and Attraction was not reliable (*β* = 0.09, 95% HDI [0.00, 0.18], *p*_+_  = 97.09%; BF_01_ = 3.45). For that reason, the interaction is not interpreted.

**Table 2:** Overview of all accuracy predicting models (1–3) for Experiment 2

Predictors	Accuracy (Median estimate of the coefficient with 95% HDI)		
	**Model 1**	**Model 2**	**Model 3**
	β (95% HDI)	β (95% HDI)	β (95% HDI)
Intercept	-0.01 [-0.10, 0.08]	-0.02 [-0.11, 0.07]	-0.02 [-0.11–0.07]
Age Group		-0.05 [-0.14, 0.04]	-0.05 [-0.15, -0.04]
Attracted to Partner			0.25 [0.16, 0.34]
Age Group × Attracted to Partner			0.09 [0.00, 0.18]
**Random Effects**			
Var(Participant)	0.00	0.00	0.00

### Discussion Experiment 2

The goal of Experiment 2 was to assess whether the low accuracy observed in Experiment 1 was the result of the simultaneous video stream used in Experiment 1. Our results are straightforward. First, we found no difference between children’s and adults’ accuracy. Further, we replicate the finding participants could not reliably detect attraction or its absence in the dating videos. Interestingly, we also replicate the effect that participants detected attraction somewhat more accurately (56%) when the person depicted was attracted to their partner than not (44%).

## Experiment 3

In Experiment 3, we manipulated the phase and length of the presented video segment. We used muted videos from the Verbal Interaction (VI) phase of Prochazkova et al.’s study (2021) and varied their lengths (i.e., 3, 6, and 9 s). Furthermore, to probe whether the observed accuracy was due to a general low emotion recognition accuracy, we included an additional Emotion Recognition Task (ERT). Low scores in the ERT would indicate that participants could not detect basic emotional expressions and might explain the low accuracy in our task of primary interest (AJT). Also, to ensure that the low accuracy was not due to potential individual differences that might influence emotion detection accuracy, we collected information using the Autism-Spectrum Quotient (AQ; Baron-Cohen et al., 2001) and Beck-Depression Inventory (BDI-II; Beck et al., 1996). Participants also indicated whether they were in a relationship or not and its duration. Because in Study 2 there were no differences between children and adults in the accuracy of detecting attraction, for feasibility, we decided to recruit adults only.

### Methods

Due to restrictions because of the COVID-19 pandemic, data collection took place online using the Gorilla platform (Anwyl-Irvine et al., 2020).

#### Participants

One hundred and seventy-six (*N* = 176) adults were recruited using social media platforms and the university psychology student pool, 13 of whom did not complete the study. Therefore, the final sample consisted of 163 participants (age range: 18–66; *M* age: 27.69, *SD* = 13.20; 95 female). All participants provided informed consent and were informed that they could withdraw their participation with no adverse consequences as according to the Declaration of Helsinki. Participants were not remunerated for their participation except for course credits. The study was approved by the Leiden University Psychology Ethics Committee (CEP 2020–02-27-M.E. Kret-V2-2192). Differences in participants’ age and gender between Experiment 1, Experiment 2, and Experiment 3 are reported in the Supplementary Material. Participants’ emotion recognition was good (75% correct) and in line with previous studies (e.g., Akdag, 2020).

#### Stimuli

Regarding the Attraction Judgment Task (AJT), to examine whether the overall low mean accuracy observed in Experiment 1 and Experiment 2 was due to either the brief duration of the stimuli or the interaction phase employed (i.e., first-impression phase; FI), in Experiment 3, we manipulated the video segment in two ways: length and interaction phase. Specifically, we used the following segments: a) 3-s FI segments (as in Experiment 1 and Experiment 2); b) 3-s; c) 6-s; and d) 9-s segments from the verbal interaction (VI) phase.

#### Experimental Task

The AJT was the same as in Experiment 2. Participants were assigned in the stimulus condition in a counterbalanced order.

#### Procedure

After participants provided informed consent, they were asked to provide demographic information (i.e., age, gender, sexual orientation, nationality, and educational level). Next, participants were informed that they would view a series of videos and, they should indicate whether the person depicted would like to go on another date with their partner and their level of certainty regarding their judgement. Participants were instructed to respond as fast and accurately as possible. Participants were prompted to indicate whether the person would like to go on another date with their partner by pressing the corresponding keyboard key (*y* yes, and *n* for no); followed by their certainty regarding their decision from 0 (*not at all*) to 6 (*very*) with 3 indicating a neutral level of certainty. Participants were prompted to take a break after 16 trials. The task consisted of 32 trials in total.

Following the AJT, participants performed the ERT (for a description of the stimuli, see Supplemental Material). Each trial started with a centrally presented fixation cross for 1000 ms, followed by the video stimulus. Then, six buttons displaying all possible emotional expressions (i.e., happy, sad, surprised, fearful, angry, neutral) were displayed and remained visible until a response was provided. Participants first practiced the task (5 trials) and then completed the task (60 trials in total). Participants were not provided feedback for their responses and were prompted to take a break after 30 trials.

After completion of the ERT, participants filled in the AQ and BDI-II and indicated if they were in a relationship, and if so its duration and qualitative status (e.g., married, dating, cohabitating). The study lasted approximately 25 min. After finishing the study, participants were debriefed and thanked for their participation.

#### Statistical Analyses

Regarding the AJT, we excluded trials with RTs < 200 ms (0.04% across all conditions). Trials on which there were technical issues, for instance regarding the presentation of the videos, were also excluded (0.16%). After applying our exclusion criteria, we were left with 99.80% of the data. Regarding the ERT, practice trials and trials with RTs faster than 200 ms were excluded (0.01%).

To model accuracy, we followed the procedure as detailed in Statistical Analyses [see General Methods section]. We coded the predictor Video Condition using a sum-contrasting scheme. All models included a random intercept per Participant. Our analyses were pre-registered on the AsPredicted database.^1^

### Results

**Fig. 6:**
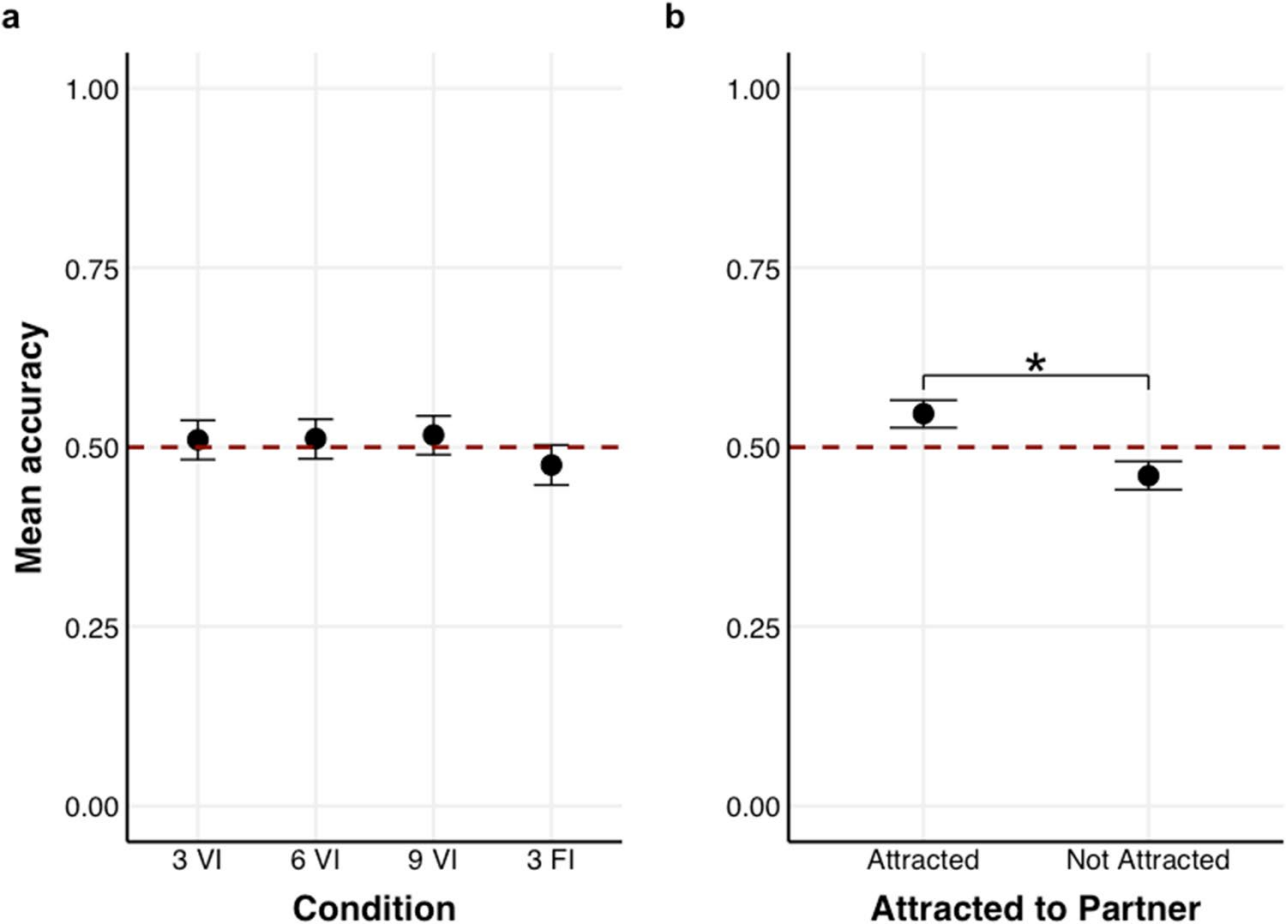
**(a)** Accuracy as a function of Video Condition (VI = Verbal Interaction; FI = First Impression (videos were muted in both conditions). Values 3, 6, 9 indicate the durations of the video segments in sec. The graph shows that people could not reliably detect attraction; **(b)** Accuracy as a function of whether the person depicted wanted to date their partner or not. For all graphs, the red dotted line denotes the chance level (0.5) and errors bars reflect 95% Credible Intervals (CrI)

First, we found no substantial evidence that participants could reliably detect attraction (*β* = 0.01, 95% HDI [-0.04, 0.07], *p*_+_  = 69.39%). After adding the fixed effect of Video Condition, there was no substantial difference in accuracy between conditions (see Fig. 6a; Table 3 Model 2); therefore, longer video segments did not influence participants’ ability to detect attraction in others.

**Table 3:** Overview of all accuracy predicting models (1–3) for Experiment 3

Predictors	Accuracy (Median estimate of the coefficient with 95% HDI)		
	**Model 1**	**Model 2**	**Model 3**
	β (95% HDI)	β (95% HDI)	β (95% HDI)
Intercept	0.01 [-0.04, 0.07]	0.01 [-0.04, 0.07]	0.01 [-0.04, 0.07]
VI3		0.03 [-0.07, 0.12]	0.03 [-0.07, 0.12]
VI6		0.03 [-0.07, 0.12]	0.03 [-0.07, 0.13]
VI9		0.05 [-0.04, 0.14]	0.05 [-0.04, 0.15]
Attracted to Partner			0.17 [0.12, 0.23]
VI3 × Attracted to Partner			-0.15 [-0.25, -0.06]
VI6 × Attracted to Partner			0.04 [-0.05, 0.14]
VI8 × Attracted to Partner			0.15 [0.05, 0.24]
**Random Effects**			
Var(Participant)	0.00	0.00	0.00

To examine whether participants can detect the presence of attraction, we added the fixed effect of Attraction to Partner and its interaction with Video Condition (Table 3 Model 3). The model showed that participants were more accurate when the person depicted wanted to date their partner than when they did not (*β* = 0.17, 95% HDI [0.12, 0.23], *p*_+_  = 100%, see Fig. 6b). Furthermore, we examined whether the interaction between Attraction to Partner and Video Condition was reliable by comparing this model to a more parsimonious model (i.e., excluding the interaction). The resulting Bayes Factor revealed that the more parsimonious model was moderately preferred over the more complex mode (BF_01_ = 6.11). Therefore, the interaction between Attraction to Partner × Video Condition is not interpreted.

#### Control analyses: performance in the Emotion Recognition task

We examined accuracy in the ERT task using an intercept-only Bayesian logistic mixed model on accuracy scores. The model showed that participants were reliably more accurate than chance level (*β* = 1.13, 95% HDI [1.06, 1.19], *p*_+_  = 100%); indicating that participants were attentive during the task and could reliably detect basic emotional expressions. Only one participant exhibited a mean accuracy below 0.5 (*M* = 0.47); excluding this participant did not change the results of our main analyses (*β* = 1.13, 95% HDI [1.06, 1.20], *p*_+_  = 100%). Thus, they were not excluded from the dataset.

### Discussion experiment 3

Our main question was whether people could accurately detect attraction. Interestingly, even when using more prolonged and more informative video segments taken from later phases of the interaction, participants were not reliably better than the chance level in detecting whether the daters were attracted to their partner or not. We also replicated the finding from experiments 1 and 2 that participants were more accurate when the person depicted was attracted to their partner than when they were not.

## General discussion

In a series of three experiments, we found no strong evidence supporting the notion that people can reliably detect attraction or its absence in thin video slices of people on a date based on nonverbal subtle emotional cues. However, we found that accuracy was increased based on whether the person presented in the video was attracted to their partner. Specifically, we found that the third-party observers were more accurate in detecting attraction when the daters were attracted to their partners than detecting the absence of attraction when the daters indicated not being attracted to their partner. In addition, recognizing attraction was not influenced by age or length of the stimuli presented.

In accordance with previous findings (e.g., Place et al., 2009), we found that people cannot reliably detect attraction from initial interactions. Given that previous findings have emphasized the importance of subtle nonverbal cues in communicating attraction (e.g., Eibl-Eiblsfeldt, 1989; Keltner & Buswell, 1997), one might question whether the observed low accuracy in detecting attraction might be the result of a low frequency of occurrence of behaviours associated with attraction. In other words, was there sufficient information present in the stimuli themselves that the participants might have picked up? Indeed, we only found minor numerical differences in behaviours associated with attraction (e.g., coyness, genuine smiles) in the First Impression 3-s videos (see Supplemental Material). Thus, the observed low accuracy might result from the low frequency of behaviour occurrence. Nonetheless, our findings replicate previous research (e.g., Place et al., 2009) and further support the notion that people cannot reliably detect attraction when viewing others in the initial phases of their interaction.

Our findings do not provide support for the notion that third-party observers can detect attraction when viewing segments from later phases of a date, which contrasts with previous research (Place et al., 2009). In all experiments, participants performed near chance level independent of the length of the segment (3, 6, or 9 s) or the phase of the interaction (first impression or verbal interaction). Our analyses (see Supplementary Material) of the coded behaviours illustrate that daters that were attracted to their partner exhibited behaviours associated with attraction for a longer duration compared to daters that were not interested in their partner (in videos taken from the middle of the speed date). This finding suggests that the observed low accuracy is not due to the low frequency of behaviour occurrence. Instead, it might be more probable that people cannot detect attraction as third-party observers using thin video slices even when the signs of attraction are there.

It may be advantageous for humans to mask what they feel in certain situations, and they often use their cognitive resources to do so (Kret, 2015). This masking might render interpreting nonverbal cues more complex and thus, lead to confusion and awkward social encounters (Abbey, 1982; Abbey & Melby, 1986) when the expressions of the sender are misinterpreted (Burgoon et al., 2002; Grammer, 1990). These factors may be a source of error in people involved in a one-on-one interaction (i.e., a date), given that the high-intensity motivational environment might decrease accurate emotion detection (Maner et al., 2005; Prochazkova et al., 2021).

It has been speculated that the ability to detect attraction in others has an adaptive function, allowing people to collect more information to guide their mating choices (see Simao & Todd, 2002). However, a more parsimonious explanation would be that the ability to detect attraction as a third-party observer is merely a by-product of detecting attraction when faced with a potential mate, which would undoubtedly be a beneficial quality for anyone navigating their romantic environment. However, previous research consistently demonstrates that people cannot detect attraction in others and instead project their interest to a given partner (Lee et al., 2020; Samara, Roth, & Kret, 2020; see also Prochazkova et al., 2021). Thus, it remains possible that people cannot detect attraction above chance level.

Emotions can be efficiently detected from facial expressions (Ekman, 1992). Previous research has shown that basic emotions, such as disgust, fear, and happiness, can be recognized in scenes within 200 ms (Righart & de Gelder, 2008). This effect suggests that detection and recognition of emotional expressions likely rely on quick facial expression processing (see also Meeren, van Heijnsbergen, & de Gelder, 2005, for similar findings on the interaction between facial expressions and body language). Here, we examined whether attraction can be detected as efficiently as other emotions. Given our null findings, we cannot conclude whether indeed attraction can be detected as efficiently as other emotions based on three experiments. Future research should help elucidate how easily and accurately complex emotions like attraction are perceived and processed.

In all experiments, we consistently found that people are likely to detect attraction when the person observed is indeed exhibiting such signals. Indeed, even though attraction cannot be expressed with a single behaviour (Moore, 1985), people likely have experience in decoding such cues and are thus more likely to detect them efficiently. This is further corroborated by our consistent replication of this effect in initial encounters as well as later in the interactions irrespective of video length (3, 6, and 9 s). Date members that were attracted to their partner likely illustrated affiliation more clearly (e.g., see Grammer et al., 1999). In contrast, disinterested partners might have opted to display rejection more subtly (or perhaps not at all), making it more challenging to interpret. However, it should be noted that we did not find robust differences in attraction cues between daters that were interested in their partner compared to daters that were not in the 3-s stimuli, even though a robust difference was found for coy smiles in the 9-s stimuli. An alternative explanation for the finding is that participants were more likely to detect attraction when indeed, participants had a general propensity to respond positively rather than negatively (see Supplemental Material). This could be due to expectancy effects, given that participants were informed that these video segments are from a blind date study. Future research should further investigate the role of expectancy effects in the ability of third-party observers to detect attraction.

This finding directly contrasts with previous research (Hall et al., 2015 Experiment 2). In their study, the authors asked participants to view 1-min segments of others on a date and indicate whether they thought the person on the video was flirting with their partner. Given that the people that report feeling attracted to their partner are also more likely to report flirting (Hall et al., 2015; Experiment 1), this is a reliable indicator of detecting attraction. Furthermore, their results suggest that participants were more accurate in detecting attraction when the person depicted was not flirting than when they were flirting. The authors suggest that these findings could be due to a) the implicit risk of openly displaying interest in another, which would have rendered any flirting difficult to decode, and b) that the probability of flirting in zero-acquaintance settings is relatively low (e.g., Abbey, 1982; Saal et al., 1989); therefore, people might not be familiar with flirting expressions in such settings. We disagree with both of these interpretations. Flirting, in general, is quite ambiguous, as flirting cues are also easily confused with friendliness (Farris et al., 2008; Moore, 2010). Furthermore, previous research has documented several flirting signals in first time-encounters, such as self-grooming (McCormick, Perper, & Jones, 1983), suggesting that these are signals typically exhibited in such situations. Crucially, in a previous study (Prochazkova et al., 2021), it was found that almost half (44%) of the participants reported that they would be interested in going on another date with their partner rendering the reduced-likelihood interpretation unlikely. In short, we consistently show that attraction is detected above the chance level when it is indeed there.

Based on the Perception–Action Model of Empathy (PAM; de Waal & Preston, 2017), we expected that participants with more experience with romantic interactions (i.e., adults) would be more accurate in detecting attraction than participants with less experience with romantic interactions (i.e., children). However, in Experiment 2, we found no substantial differences between adults and children, suggesting that children’s lower accuracy in detecting attraction in Experiment 1 was likely due to cognitive overload.

One limitation that should be discussed is the fact that our responses were coded in a binary way. This approach was necessary to calculate accuracy based on the responses of the study conducted by Prochazkova et al. (2021), where responses were also coded binary. It could be argued that this approach reduced the variation that would otherwise be shown if responses were coded in a continuous way. This is indeed possible, even though it should be noted that using a scale for attraction and a binary response for another date has been shown to correlate highly (Roth et al., 2021a, 2021b). Nonetheless, future studies using speed-dating paradigms could also employ a continuous response regarding attraction and willingness to go on another date, which can then be used in studies employing third-party observers. In this manner, a more nuanced accuracy scale can be calculated.

In conclusion, here we demonstrate that people might not reliably detect when others are attracted to their partner and when not. Furthermore, we showed that the overall accuracy in detecting attraction is not influenced by age, or the phase of the interaction observed. The only factor that reliably influenced accuracy is whether attraction is indeed present.

## Supplementary Information

Below is the link to the electronic supplementary material.

Supplementary file1 (DOCX 2.55 MB)

## Footnotes

1 Reference number: #37,849.
