# Supplementary material for: Can third-party observers detect attraction in others based on subtle nonverbal cues?
Source: Curr Psychol. 2022 Apr 8:1–15. Online ahead of print. doi: 10.1007/s12144-022-02927-0 (PMC8990491; doi:10.1007/s12144-022-02927-0)
Supplement: Supplementary file 1 — Supplementary file1 (DOCX 2.55 MB) [file 12144_2022_2927_MOESM1_ESM.docx]

**Supplemental material**

**Expressions of attraction in videos**

The video segments were coded to examine whether daters exhibited expressions signalling attraction during the dates. All video segments (*N* = 32; 16 female) were coded offline using the Observer XT 11.5 event-logging software (Noldus, Trienes, Hendriksen, Jansen, & Jansen, 2000) for both the 3-second First Impression (FI) and 9-second Verbal Interaction (VI) conditions, for a total of 64 videos. We used a coding scheme including multiple composite and single-unit behaviours associated with positive experience during romantic interactions. Specifically, we coded the following expressions: a) coyness; b) flirting; c) interest; d) positive affect; e) embarrassment, and minor variations of these expressions thereof (e.g., coy smiles with and without raised cheeks; see Table 1) based on Cordaro et al. (2018). The video segments were coded by two independent coders following extensive training. To assess inter-rater reliability, Cohen’s intra-class correlation (ICC) for absolute agreement was calculated for 4 video segments (12.5% percent of video segments; IIC > 0.60, for all continuous behaviours, except embarrassment which was not included in the analyses and kappa = 1.00 for all categorical behaviors). The durations and frequencies were extracted and analysed in JASP (version 0.16; JASP Team, 2021). For all coded behaviours, we compared daters who were interested to their partner versus daters who were not interested in their partner. We used either independent Bayesian *t*-tests or chi square tests for continuous and count data, respectively. All tests were conducted using default prior distributions.

Table 1.

| *Description of coded behaviours including Event Type, Description, and associated Reliability Values (IIC for absolute agreement or Cohen’s kappa)* | | | |
| --- | --- | --- | --- |
| Variable | Event type | Description | Reliability |
| Leaning forward | Event | Leaning toward the partner with the upper body | 1 |
| Coyness | State | A smile (lip corners raised) in combination with a gaze aversion | 0.99 |
| Coyness (cheeks raised) | State | A genuine smile (lip corners raised; cheeks raised) in combination with a gaze aversion | 0.94 |
| Flirting | State | A smile (lip corners raised) with eye-contact and head tilt | 0.69 |
| Head nod | State | Nodding | 1 |
| Genuine smile | State | Lip corners up with cheeks raised | 0.76 |
| Polite smile | State | lip corners up without cheeks raised | 0.86 |
| Blushing | Event |  | 1 |
| Rolling the pelvis | Event |  | 1 |
|  |  |  |  |

Regarding the 3 second videos, there were no robust differences in expression duration between daters that were interested in their partner and daters that were not interested in their partner. However, there were numerical trends between conditions (see Table 2) suggesting that the number of videos might have limited our power to detect these differences.

Regarding the 9 second videos, daters showed more coyness when they were attracted to their partner (*M* = 2740.00 ms; *SD* = 1137.52) than when they were not (*M* = 1700.00 ms; *SD* = 916.52; BF_10_ = 3.13; see Table 3). All other expressions had a BF_10_ < 3 (indicating anecdotal evidence) and thus were not interpreted (BF_10_: coyness (with cheek raised) = 0.54; flirting = 1.47; genuine smile = 0.35; polite smile = 0.58). Bayesian contingency tables showed no robust differences in head nodding, blushing, and rolling the pelvis (coded 1 if present; 0 if absent) between daters who were interested in their partner than not (BF_10_: blushing = 0.43; rolling the pelvis = 0.59).

| Table 2.  *Overview of differences in duration of emotional expressions between videos in which the daters were attracted*  *to their partner or not in First Impression 3-second videos.* | | | | | | | | |
| --- | --- | --- | --- | --- | --- | --- | --- | --- |
|  | **Attracted to Partner** | | |  | **Not Attracted to Partner** | | |  |
| Behavior | *M* | *SD* | 95% CrIs |  | *M* | *SD* | 95% CrIs | *BF*_10_ |
| Coyness cheek raised | 293.75 | 392.38 | 84.67, 502.83 |  | 443.75 | 847.72 | -7.97, 895.47 | 0.39 |
| Coyness | 800.00 | 752.77 | 398.88, 1201.12 |  | 693.75 | 813.61 | 260.21, 1127.29 | 0.36 |
| Genuine smile | 1175.00 | 1216.28 | 526.89, 1823.11 |  | 618.75 | 1059.07 | 54.41, 1183.09 | 618.75 |
| Polite smile | 1562.50 | 1071.37 | 991.61, 2133.39 |  | 2081.25 | 1366.37 | 1353.17, 2809.34 | 0.58 |
| Flirting | 4 |  |  |  | 12 |  |  | 0.52 |
| Blush | 6 |  |  |  | 10 |  |  | 0.53 |
| Rolling pelvis | 0 |  |  |  | 1 |  |  | 1.03 |

Note: Flirting was treated as categorical due to the low variance in duration.

| Table 3.  *Overview of differences in duration of emotional expressions between videos in which the daters were attracted*  *to their partner or not in 9-second videos.* | | | | | | | | |
| --- | --- | --- | --- | --- | --- | --- | --- | --- |
|  | **Attracted to Partner** | | |  | **Not Attracted to Partner** | | |  |
| Behavior | *M* | *SD* | 95% CrIs |  | *M* | *SD* | 95% CrIs | *BF*_10_ |
| Coyness cheek raised | 1546.67 | 1326.70 | 792.03, 2301.31 |  | 1081.25 | 977.90 | 560.16, 1602.34 | 0.54 |
| Coyness | 2740.00 | 1385.02 | 1973.00, 3507.00 |  | 1700.00 | 916.52 | 1211.62, 2188.38 | 3.13 |
| Genuine smile | 2833.33 | 1776.30 | 1849.65, 3817.02 |  | 2643.75 | 2275.95 | 1430.98, 3856.52 | 0.35 |
| Polite smile | 2683.33 | 1831.30 | 761.50, 4605.17 |  | 3580.00 | 2025.29 | 2131.20, 5028.80 | 0.58 |
| Flirting | 446.67 | 552.74 | 140.57, 752.77 |  | 1093.75 | 1137.52 | 487.61, 1699.89 | 1.47 |
| Blush | 6 |  |  |  | 10 |  |  | 0.43 |
| Rolling pelvis | 3 |  |  |  | 2 |  |  | 0.59 |

**Participant accuracy per actor**

Here, we explored whether participants were more likely to detect attraction in some videos than others. We plotted the mean accuracy in detecting attraction by Actor (person depicted in the video; see Figure 1). Interestingly, it can be noted that 10 videos (2 women; 7 attracted to partner) were consistently rated with over 0.5 level accuracy (henceforth referred to as *conspicuous*) compared to all other videos in our stimulus set (henceforth referred to as inconspicuous). This pattern suggests that these video segments might have specific elements that rendered them easier to interpret, such as higher duration of behaviours associated with attraction.

To investigate this question, we split the data between the Conspicuous (*N* = 10) and Inconspicuous videos (*N* = 22) and analysed whether the duration of behaviours associated with attraction differed between these two groups (for an overview of all descriptives see Table 3). Bayesian independent *t*-tests showed that in Conspicuous videos, daters exhibited a greater duration of happiness (*M* = 1540.00, *SD* = 1200.19) compared to all remaining videos (*M* = 604.55, *SD* = 1035.32; BF_10_ = 2.20). Furthermore, in the Conspicuous videos, daters exhibited lower duration of polite smiles (*M* = 1040.00; *SD* = 915.55) compared to the Inconspicuous videos (*M* = 2177.27; *SD* = 1213.78; BF_10_ = 4.09).


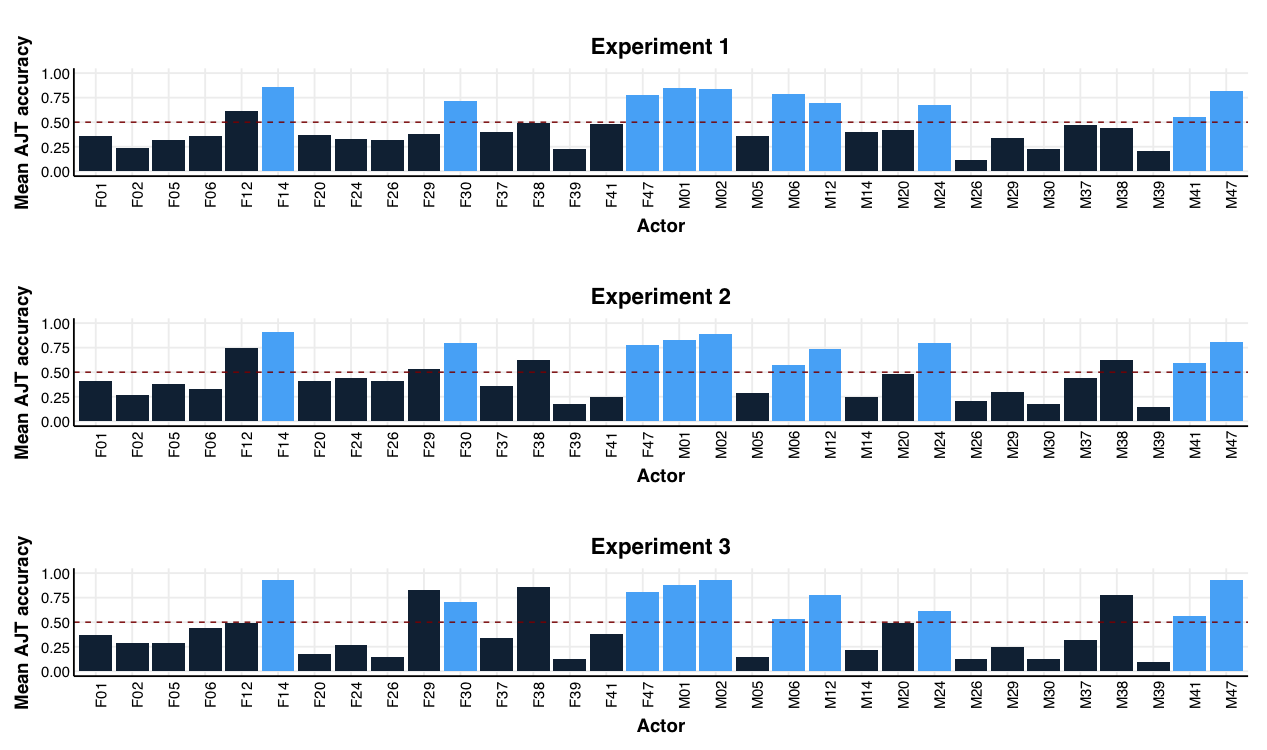


*Figure 1.* Mean accuracy as a function of Actor (person depicted in video) for Experiments 1-3. Only accuracy from the FI3 Condition are presented in all experiments. The red line denotes chance level (0.5) accuracy. Stimuli that were consistently rated with over 0.5 accuracy are presented in light blue.

| Table 3.  *Overview of differences in duration of emotional expressions between Inconspicuous and Conspicuous videos* | | | | | | | | | |
| --- | --- | --- | --- | --- | --- | --- | --- | --- | --- |
|  | **Inconspicuous** | | | |  | **Conspicuous** | | |  |
| Behavior | *M* | | *SD* | 95% CrIs |  | *M* | *SD* | 95% CrIs | *BF*_10_ |
| Coyness cheek raised | 345.46 | | 740.481 | 17.14, 673.77 |  | 420.00 | 436.654 | 107.64, 732.36 | 0.37 |
| Coyness | 872.73 | | 864.20 | 489.56, 1255.89 |  | 470.00 | 437.29 | 157.18, 782.82 | 0.72 |
| Genuine smile | 604.545 | | 1035.318 | 145.51, 1063.58 |  | 1540.00 | 1200.19 | 681.44, 2398.56 | 2.20 |
| Polite smile | 2177.27 | | 1213.783 | 1639.11, 2715.43 |  | 1040.00 | 915.545 | 385.06, 1694.94 | 4.09 |
|  |  |  | | | | | | | |

**Positive response bias**

Here, we examined whether differences in attraction detection accuracy as a function of whether the dater depicted was attracted to their partner or not can be explained by a general propensity of the participants to indicate that a dater is attracted to their partner more often than that a dater is not attracted to their partner. We conducted three Bayesian Generalized linear mixed models with participant response (yes/no) as dependent variable and Attraction to Partner as a fixed effect. All models included a random intercept per participant (nested in Group ID for Experiment 1).

The results show that participants were indeed more likely to generally respond yes than no (Exp 1: *β* = 0.37, [0.27, 0.48], *p*_+_ = 100%; Exp 2: *β* = 0.23, [0.13, 0.33], *p*_+_ = 100%; Exp 3: *β* = 0.18, [0.10, 0.26], *p*_+_ = 100%). General response propensity was not influenced by Attraction to Partner (Exp 1: *β* = -0.06, [-0.12, 0.01], *p*_-_ = 96.28%; Exp 2: *β* = -0.01, [-0.09, 0.08], *p*_-_ = 57.26%; Exp 3: *β* = 0.01, [-0.04, 0.07], *p*_+_ = 68.91%).

**
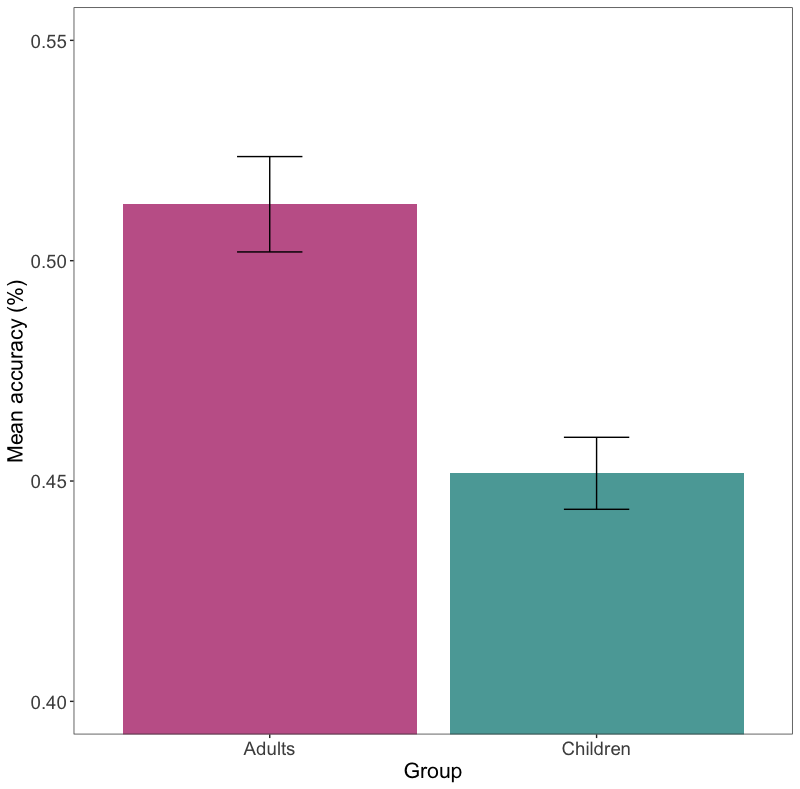
Effect of gender congruence on the detection of attraction**

To examine whether gender congruence (i.e., a match between the gender of the observer and the person observed) facilitates attraction detection, we included the fixed effects of Age Group (Experiments 1 and 2) or Video Condition (Experiment 3), respectively, Shuffled, and Gender Congruence, as well as their interaction. The analysis was conducted separately for each experiment (see Table 4). We found no substantial evidence that gender congruence facilitated attraction detection.

| **Table 4** | | | | | | | |
| --- | --- | --- | --- | --- | --- | --- | --- |
| *Overview of all Gender Congruency models for Experiments 1-3.* | | | | | | | |
| *Predictors* | *Accuracy (Median estimate of the coefficient with 95% HDI* | | | | | | |
|  | **Model 1** | | **Model 2** | | **Model 3** | |  |
|  | *β* (95% HDI) | | *β* (95% HDI) | | *β* (95% HDI) | |  |
| Intercept | -0.06 | -0.13, 0.01 | -0.02 | -0.11, 0.07 | 0.01 | -0.04, 0.07 |  |
| Age Group | -0.14 | -0.21, -0.07 | -0.05 | -0.14, 0.04 |  |  |  |
| Shuffled | -0.01 | -0.07, 0.06 |  |  |  |  |  |
| Gender Congruence | 0.02 | -0.04, 0.08 | -0.01 | -0.10, 0.09 | -0.02 | -0.07, 0.04 |  |
| VI3 |  |  |  |  | 0.03 | -0.07, 0.12 |  |
| VI6 |  |  |  |  | 0.04 | -0.06, 0.13 |  |
| VI9 |  |  |  |  | 0.05 | -0.04, 0.14 |  |
| Age Group × Shuffled | 0.04 | -0.02, 0.11 |  |  |  |  |  |
| Age Group × Gender Congruence | -0.01 | -0.07, 0.06 | 0.01 | -0.07–0.10 |  |  |  |
| Shuffled × Gender Congruence | 0.02 | -0.04, 0.09 |  |  |  |  |  |
| Age Group × Shuffled × Gender Congruence | 0.06 | 0.00, 0.12 |  |  |  |  |  |
| VI3 × Gender Congruence |  |  |  |  | 0.01 | -0.08, 0.11 |  |
| VI6 × Gender Congruence |  |  |  |  | -0.05 | -0.15, 0.04 |  |
| VI9 × Gender Congruence |  |  |  |  | 0.07 | -0.02, 0.17 |  |
|  |  |  |  |  |  |  |  |
| **Random Effects** | | | | |  |  |  |
| Var(Participant) | 0.00 |  | 0.00 |  | 0.00 |  |  |
| Var(GroupID) | 0.00 |  |  |  |  |  |  |

**Differences in sample characteristics between Experiments 1, 2, and 3.**

A Bayesian independent samples *t*-test showed no differences in age between children in Experiment 1 and Experiment 2 (BF_01_ = 3.13). A Bayesian chi-square test

showed that there were no differences in gender distribution between Experiment 1 and Experiment 2 (BF_01_ = 3.39).

A Bayesian one-way Analysis of Variance (ANOVA) showed that there were differences in age between adults (BF_10_ > 10). Specifically, the age mean in Experiment 2 was higher than Experiment 1 (BF_10_ > 10) and Experiment 3 (BF_10_ > 10). There were no differences in age between Experiment 1 and Experiment 3 (BF_01_ = 0.23).

Bayesian chi-square tests showed no differences in gender distribution between experiments (Experiments 1-2: BF_10_ = 0.58; Experiments 1-3: BF_10_ = 0.50; Experiments 2-3: BF_10_ = 0.23).

**Stimuli employed in Emotion Recognition Task**

Regarding the Emotion Recognition Task (ERT), we used stimuli from the Facial Expressions and Emotion Database (FEEDTUM; Wallhoff et al., 2006). The FEEDTUM database consists of 18 individuals displaying 7 spontaneously elicited emotional facial expressions (happiness, disgust, anger, fear, sadness, surprise, and neutral). Here, we only included 10 actors (5 female) and opted to not include the emotion of disgust. Therefore, the final stimulus set consisted of 60 videos (6 emotional expressions × 10 actors). To ensure potential luminance confounds, the background of all videos was standardized (*r* = 128, *g* = 128, *b* = 128; Akdag, 2020). All videos were 2000 ms in length, whereby the first 500 ms consisted of a neutral expression and 1500 ms of an emotional expression.

References

Akdag, R. (2020). *Emotion processing in social anxiety: An impairment in emotion recognition or self-evaluation?* (Master’s thesis, Leiden University, Leiden, the Netherlands).

Cordaro, D. T., Sun, R., Keltner, D., Kamble, S., Huddar, N., & McNeil, G. (2018). Universals and cultural variations in 22 emotional expressions across five cultures. *Emotion, 18*(1), 75-93. https://doi.org/10.1037/emo0000302

Wallhoff, F., Schuller, B., Hawellek, M., & Rigoll, G. (2006). Efficient recognition of authentic dynamic facial expressions on the Feedtum database. *2006 IEEE International Conference on Multimedia and Expo*, 493–496. https://doi.org/10.1109/ICME.2006.262433
